# Editorial: Ocular neurodegenerative diseases: novel mechanisms, diagnosis, and therapeutic strategies

**DOI:** 10.3389/fnins.2023.1274778

**Published:** 2023-09-01

**Authors:** Wenyi Wu, Yuanjun Li, Tu Hu, Wensi Tao, Dan Wen, Hetian Lei

**Affiliations:** ^1^Ophthalmology of Xiangya Hospital, Central South University, Changsha, China; ^2^Sylvester Comprehensive Cancer Center, University of Miami, Miami, FL, United States; ^3^Department of Ophthalmology, The Third Affiliated Hospital of Xinxiang Medical University, Xinxiang, China; ^4^Shenzhen Eye Hospital, Jinan University, Shenzhen Eye Institute, Shenzhen, China

**Keywords:** neurodegeneration, high myopia, glaucoma, retinopathy, macular degeneration

Ocular neurodegeneration including high myopia, glaucoma, macular degeneration, optic nerve atrophy, and retinopathy can lead to blindness without timely and appropriate treatment. As the retina is actually an extension of the brain, studies on the molecular mechanisms by which these eye diseases develop are currently one of the hottest research areas in neuroscience.

Investigations on neuronal correction of visual deficits have enriched our knowledge of functional eye diseases including ocular neurodegeneration. Nevertheless, it is urgently needed to further our understanding of how these eye diseases develop. Notably, novel therapies with superstar pharmacological intervention or new methods such as gene therapy or stem cell therapy have been attracted into this research area. The aim of this Research Topic “*Novel mechanisms, diagnostic and therapeutic strategies for ocular neurodegeneration*” is to extend our knowledge related to retina and vision disorders by bringing together work in ophthalmology, optometry, psychology, neuroscience, and vision science.

Molcak et al. have summarized the expression, distribution, functions, and interactions of purinergic receptors in the retina and included potential crosstalk with other systems. Dissection of how these processes are affected will improve our understanding of the mechanisms that drive age-related macular degeneration (AMD).

Avrutsky et al. have shown their original research data that caspase-9 inhibition has significant retinal protection from retinal vein occlusion (RVO). To be more specific, they have compared the therapeutic effect of caspase-9 inhibition with VEGF neutralization in an established mouse model of RVO, and they have conducted a series of examinations, including fundus angiography, optical coherence tomography (OCT), and electroretinography (ERG), for analyzing pathological changes.

Ye et al. have investigated the long-term safety, efficacy, and binocular balance of monovision surgery using Implantable Collamer Lens (ICL) V4c implantation and Femtosecond Laser-Assisted *in situ* Keratomileusis (FS-LASIK) for the treatment of myopic patients with presbyopia. The results show that CL V4c implantation and FS-LASIK monovision treatment have long-term safety and binocular visual acuity at various distances.

Lan et al. have demonstrated the control ability and characteristics of fixational displacement among healthy adults in a convenient method by using eye-tracking technology. A total of 100 healthy people were recruited for this study, providing an objective view that the fixation stability decreased significantly in the group aged over 50 years old.

Chen et al. have reported a non-invasive diagnosis tool to assess blood flow perfusion in a visual pathway for ocular ischemic syndrome (OIS), which is attributable to chronic hypoperfusion caused by marked carotid stenosis. They have detected blood flow perfusion in a visual pathway by 3D pseudocontinuous ASL (3D-pCASL) using 3.0T MRI (magnetic resonance imaging). The results indicate that that there is a lower blood flow perfusion value in the visual pathway in patients with OIS.

Velmurugan et al. have summarized different gene therapy methods in Leber hereditary optic neuropathy (LHON), suggesting that a mitochondrially targeted AAV (adeno-associated virus) gene therapy is more efficient than an allotopic AAV gene therapy for rescuing the LHON phenotype.

Zheng et al. have presented a surprising treatment for restoring vision in adult amblyopia rats. By using molecular and histological approaches, they have revealed that low-frequency repetitive transcranial magnetic stimulation (rTMS) reinstates the amplitude of visual evoked potentials without influencing the impaired depth perception of amblyopic rats. They conclude that rTMS enhances functional recovery and visual plasticity in an adult amblyopic animal model.

Zhang et al. have given a bibliometric analysis of apoptosis in glaucoma. This research will broaden our comprehension about the role of apoptosis in the process of glaucoma and provide guidelines for us in basic research and disease treatment.

Zhen et al. have summarized rhodopsin-associated retinal dystrophy and its disease mechanisms and therapeutic strategies. In particular, they emphasize that innovative therapy strategies, such as gene therapy (including gene editing, neuroprotection, and optogenetics) and stem cell therapy, are promising methods for the future treatment of retina pigmentosa. Nevertheless, greater efforts are needed from basic researchers and clinicians to facilitate the translation of recent research findings from the laboratory into clinical practice.

Xiang et al. have interrogated the question of whether children with monocular myopia need to wear glasses. They consider the facts that (1) monocular myopia could lead to the accommodative dysfunction and unbalanced input of binocular visual signals, resulting in myopia progression; (2) monocular myopia may also be accompanied by stereopsis dysfunction, and long-term uncorrected monocular myopia may worsen stereopsis acuity in adulthood; (3) patients with monocular myopia could exhibit stereopsis dysfunction at an early stage; thus, they come to a conclusion that children with monocular myopia must wear glasses to restore binocular balance and visual functions, thereby delaying myopia progression.

Boal et al. have presented a study showing that retinal ganglion cells adapt to ionic stress in experimental glaucoma. Their data indicate that in response to prolonged IOP (intraocular pressure) elevation, RGCs (retinal ganglion cells) undergo an adaptive process that reduces sensitivity to changes in K+ while diminishing excitability. These experiments give insight into the RGC response to IOP stress and lay the groundwork for mechanistic investigation into targets for neuroprotective therapy.

Li et al. have systemically analyzed independent risk factors for the progression of different degrees of diabetic retinopathy and non-diabetic retinopathy among type 2 diabetic patients. They conclude that young age, short axial length, and higher levels of FBG (fasting blood glucose) and urinary albumin creatinine ratio (UACR) were the independent risk factors for the progression of diabetic retinopathy in type 2 diabetes.

Wareham et al. have presented data on collagen mimetic peptide repair of the corneal nerve bed in a mouse model of dry eye disease. Their data suggest that repair of underlying collagen in conditions that damage the ocular surface could represent a novel therapeutic avenue in treating a broad spectrum of diseases or injury.

Wang and Wang have shown a preliminary study of spectral-domain optical coherence tomography (OCT) combined with ERG in the assessment of conbercept for neovascular age-related macular degeneration (nAMD). Their data suggest that (1) conbercept is useful for the short-term treatment of nAMD; (2) it can safely improve the visual acuity of affected eyes; and (3) it can restore the structure and function of the retina.

Zhou et al. have shown that increased intraocular inflammation in retinal vein occlusion is independent of circulating immune mediators and is involved in retinal oedema. Their results suggest that (1) intraocular inflammation in RVO is driven primarily by local factors but not circulating immune mediators; (2) intraocular inflammation may promote macular oedema through the PI3K-Akt, Ras, MAPK, and Jak/STAT signaling pathways in RVO; and (3) systemic factors, including cytokines and lipid levels, may be involved in retinal microvascular remodeling.

Wang et al. have assessed the precision and reliability of a novel computerized heterophoria test (CHT). Their data suggest that (1) the CHT can be used to demonstrate excellent inter- and intra-examiner repeatability and good correlation with the POCT (prism-neutralized objective cover test); (2) the differences between the CHT and POCT are within the permissible range of error; and (3) the CHT could provide a precise and reliable measurement for clinical applications.

Altogether, this Research Topic of articles emphasizes novel diagnostic and therapeutic approaches for a variety of ocular neurodegeneration diseases, which are necessary to further explore.

## Author contributions

WW: Funding acquisition, Writing—original draft. WT: Writing—review and editing. YL: Writing—review and editing. TH: Funding acquisition, Writing—review and editing. DW: Writing—review and editing. HL: Conceptualization, Funding acquisition, Writing—original draft, Writing—review and editing.

